# THE IMPACT OF SEXUAL FLUIDITY AND SOCIAL SUPPORT ON EGO INTEGRITY IN OLDER ADULTS

**DOI:** 10.1093/geroni/igz038.1117

**Published:** 2019-11-08

**Authors:** Alicia C Figueroa

**Affiliations:** Northeastern Illinois University, Chicago, Illinois, United States

## Abstract

The population of lesbian, gay, and bisexual (LGB) Americans is growing as the number of older adults “come out. ” While we know that “coming out” later in life impacts the experience of aging, little research has examined ways in which social support and sexual fluidity influence ego integrity in older adults. The present study investigated ego integration, changed sexual behavior, and perceived social support in adults 45 years of age and older who had been in a long-term relationship with the opposite sex prior to “coming out” as LGB. The average of participants was 61 years, sixty-eight percent (N = 43) were currently married or in a domestic partnership, and eighty-six percent (N = 54) identified as Caucasian. Individuals were recruited to participate in the survey utilizing online social media. Results (N = 63) suggested that those whose behavior was more sexually fluid were least ego integrated. Timing of “coming out” LGB, Early (44 and younger) versus Late (after the age of 45) impacted the degree of sexual fluidity and perceived social support. Results indicated those who “came out” Late were more sexually fluid and were less fearful of “coming out. ” The most significant result revealed social support from significant others to be most impactful compared to friends and family. This research expands on the challenges of those who are aging in a non-normative environment. The implications suggest that individuals who “come out” later have a more difficult time with accepting their sexual fluidity.

